# Steroid-induced osteonecrosis of the femoral head reveals enhanced reactive oxygen species and hyperactive osteoclasts

**DOI:** 10.7150/ijbs.40917

**Published:** 2020-04-06

**Authors:** Kai Chen, Yuhao Liu, Jianbo He, Nathan Pavlos, Chao Wang, Jacob Kenny, Jinbo Yuan, Qingwen Zhang, Jiake Xu, Wei He

**Affiliations:** 1 School of Biomedical Sciences, The University of Western Australia, Perth, WA 6009, Australia; 2 Department of Joint Orthopaedics, The First Affiliated Hospital, Guangzhou University of Chinese Medicine, Guangzhou, Guangdong 510405, China; 3 The Lab of Orthopaedics of Chinese Medicine, Lingnan Medical Research Center, Guangzhou University of Chinese Medicine, Guangzhou, Guangdong 510405, China; 4 Research Institute of Orthopaedics of Chinese Medicine, Guangzhou University of Chinese Medicine, Guangzhou, Guangdong 510378, China

**Keywords:** Reactive oxygen species (ROS), Osteonecrosis of femoral head (ONFH), Osteoclasts, Antioxidant enzymes

## Abstract

Steroid-induced osteonecrosis of the femoral head (ONFH) is a progressive bone disorder which typically results in femoral head collapse and hip joint dysfunction. It is well-accepted that abnormal osteoclast activity contributes to loss of bone structural integrity and subchondral fracture in ONFH. However, the pathophysiologic mechanisms underlying the recruitment and hyperactivation of osteoclasts in ONFH remain incompletely understood. We assessed the changes of reactive oxygen species (ROS) level and subsequent osteoclast alterations in steroid-induced osteonecrotic femoral heads from both patients and rat ONFH models. When compared with healthy neighboring bone, the necrotic region of human femoral head was characterized by robust up-regulated expression of osteoclast-related proteins [cathepsin K and tartrate-resistant acid phosphatase(TRAP)] but pronounced down-regulation of antioxidant enzymes (catalase, γ-glutamylcysteine synthetase [γ-GCSc], and superoxide dismutase 1 [SOD1]). In addition, the ratio of *TNFSF11* (encoding RANKL)/*TNFRSF11B* (encoding OPG) was increased within the necrotic bone. Consistently, in rat ONFH models induced by methylprednisolone (MPSL) and imiquimod (IMI), significant bone loss in the femoral head was observed, attributable to increased numbers of TRAP positive osteoclasts. Furthermore, the decreased expression of antioxidant enzymes observed by immunoblotting was accompanied by increased *ex-vivo* ROS fluorescence signals of dihydroethidium (DHE) in rat ONFH models. Therefore, this study lends support to the rationale that antioxidant agents may be a promising therapeutic avenue to prevent or mitigate the progression of steroid-induced ONFH by inhibiting ROS level and hyperactive osteoclasts.

## Introduction

Osteonecrosis of the femoral head (ONFH) is a progressive degenerative disorder of the hip characterized by subchondral bone microfractures and subsequent collapse of the femoral head, eventually leading to dysfunction of the hip joint 1. ONFH poses a huge socioeconomic burden, usually affecting young to middle aged individuals, with ~67% of asymptomatic patients rapidly progressing to symptomatic stages and thus requiring total hip arthroplasties (THA) 2. The exact aetiology of ONFH is unclear, however, steroid administration is an established risk factor, which accounts for ~51% of all reported ONFH cases 3. For example, in patients who received corticosteroid treatment for systemic lupus erythematosus (SLE), it was reported that an increase of corticosteroids to 10 mg/d correlated with a 3.6 % increase in the rate of osteonecrosis, with daily doses >40 mg predisposing patients to ONFH 4. Consistently, patients with severe acute respiratory syndrome (SARS) can also develop ONFH and progress into late stage following steroid administration 5. The other known contributors include alcohol abuse, tobacco use, coagulation abnormalities, chemotherapy, genetic factors, and idiopathic cause 3, 6, 7.

The pathophysiologic mechanisms underlying steroid-induced ONFH are not fully understood. Recent studies suggest that the onset of ONFH is initiated by impaired microcirculation and necrosis of osteocytes, with magnetic resonance imaging (MRI) detecting pools of edema 1, 8, but without noticeable changes in subchondral bone microstructure, as assessed by computed tomography (CT). Following bone necrosis, a repair process ensues whereby osteoclast-mediated bone resorption surpasses bone formation by osteoblasts, leading to net loss of subchondral trabecular bone 9. As the ONFH progresses, the loss of bone and altered microarchitecture compromises the integrity of the femoral head resulting in deformity and high vulnerability of collapse 8. It is widely accepted that excessive osteoclast activity during the regenerative phase, rather than during the necrosis of cells and tissue, directly contributes to the loss of bone integrity and ensuing subchondral bone fracture 8-11. This contribution is further supported by studies showing that anti-resorptive agents could effectively reduce the risk of femoral head collapse at early-stage ONFH when compared with placebo treatment 12-14.

A better understanding of the activation of osteoclasts during repair process is therefore critical in order to develop effective treatments to preserve the integrity of hip joint prior to femoral head deformation and collapse. Accumulated evidence indicate that reactive oxygen species (ROS) play a crucial role in osteoclast formation and function by modulating receptor activator of NF-κB ligand (RANKL)-induced signalling 15-17. Our previous work has demonstrated that ROS levels are increased during estrogen deficiency-induced osteoporosis in mice, and that antioxidant agents are effective in inhibiting osteoclast activity and thus preventing bone loss 18. ROS have also been implicated in the pathogenesis of ONFH, causing damage to blood vessels and osteoblastic cell lineages 19-21. However, the underlying mechanisms by which ROS levels change and cause the activation of the osteoclasts in the context of steroid-induced ONFH remain poorly understood. Our study revealed that the high oxidative stress following steroid administration may result from decreased expression of antioxidant enzymes, eventually leading to osteoclast hyperactivity and subsequently ONFH progression.

## Methods

### Specimens Collection

Steroid-induced osteonecrotic femoral heads (ARCO stage III-IV) were collected from three patients who received total hip arthroplasty (THA) at the First Affiliated Hospital of Guangzhou University of Chinese Medicine (Guangzhou, China). All three patients with ONFH had a medical history of the steroid administration owing to different primary diseases - systemic lupus erythematosus (SLE), fever of unknown origin (FUO), and rheumatic arthritis. This study was approved by the Ethical Committee of the First Affiliated Hospital of Guangzhou University of Chinese Medicine (No. Y [2019] 141). Informed content was obtained from the patients included in this study.

### Steroid-induced ONFH rat Model

Male Sprague Dawley (SD) rats at 10-weeks-of-age were provided by Sanyuanli Animal Experiment Center of Guangzhou University of Chinese Medicine (Guangzhou, China). All experimental procedures in this study were approved by the Institutional Animal Ethics Committee of Guangzhou University of Chinese Medicine (NO.20190213001). All rats were housed under a 12-hour light/dark circle and had access to water and food ad libitum. Animals were randomly divided into two groups: control group (n=8) and rat ONFH model group (n=8). Steroid-induced ONFH models were implemented as previously described 22, 23 with modifications. Rats in the ONFH model group were given Imiquimod (IMI, 30 mg/kg) subcutaneously on day 1 and Methylprednisolone (MPSL, 20 mg/kg) intramuscularly on day 2. All injections were repeated on week 4. Rats in the control group were injected with the same amount of saline at the same time point with the model group. Rats were sacrificed and the femurs were collected at 6 weeks following the first injection. Dihydroethidium (DHE, 20 mg/kg) was subcutaneously injected 24 hours prior to sacrifice to visualize the ROS fluorescence signals of DHE 18.

### Histomorphometric analysis

Rat femurs or human femoral head regions of interest were collected. The overlying tissues were removed prior to the fixation in 4% paraformaldehyde (PFA) for 48 hours. Bone tissues were decalcified in EDTA (14%, PH=7.4) (Sigma-Aldrich) at 37 °C for 14 days, in which the EDTA solution was changed every day till the bone became soft for sectioning. Next, the bone samples were placed in the automatic tissue processor for dehydration, followed by paraffin-embedding. Sections of 5-μm thickness were cut using a Leica RM 2155 Biocut Microtome (Leica Microsystems) and collected onto glass slides. Haematoxylin and eosin (HE) staining and tartrate-resistant acid phosphatase (TRAP) staining were implemented to visualise the bone microstructures and osteoclasts. Stained and mounted bone sections were scanned with Uscope MXII-20 (ProSciTech). Osteoclast parameters including osteoclast number per bone surface (N.Oc/BS) and osteoclast surface per bone surface (Oc.S/BS) were analysed using Bioquant Osteo software (Bioquant Image Analysis Corporation).

### *In-vivo* ROS fluorescence detection

The cryosections of rat femoral heads were prepared as described previously 18 with modifications. Freshly isolated rat femoral heads were fixed using 4% PFA at 4 °C for 48 hours, which was followed by the decalcification process using EDTA (0.5 M, PH=7.4) for 4 weeks at 4 °C under gentle rocking. Next, the samples were cryo-protected by incubating in solutions of 2% polyvinylpyrrolidone (PVP) and 20% sucrose for 24 hours at 4°C. The resultant tissues were embedded in Tissue-Tek optimum cutting temperature (O.C.T.) compound (Tissue-Tech). A thickness of 5-μm sections was obtained at a low speed of cutting and then collected using slides, followed by air-dry at room temperature for 30 minutes. DAPI solution was used to stain the nuclei prior to mounting using ProLong Gold Antifade Mountant (Thermo Fisher Scientific). The images were observed using an A1Si confocal microscope (NIKON). Appropriate laser settings were chosen based on the excitation/emission wavelengths of the desired fluorochrome (DAPI, 358nm/461 nm; DHE, 490nm /590 nm) and the regions of interest were captured.

### Quantitative real-time polymerase chain reaction (qPCR)

Total RNA was extracted from the bone tissue using TRIzol reagent (Life Technologies) and an RNA Extraction Kit (TaKaRa) following the manufacturer's instructions. The extracted RNA was transcribed in complementary DNA (cDNA) using reverse transcriptase with OligodT primer and moloney murine leukemia virus (MMLV) (Promega). The primers used in this study include *TNFSF11*, encoding receptor activator of nuclear factor kappa-Β ligand (RANKL); *TNFRSF11B*, encoding osteoprotegerin (OPG); *CTSK*, encoding cathepsin K; *ACP5*, encoding TRAP; *CAT*, encoding catalase; *NQO1*, encoding NAD(P)H quinone dehydrogenase 1; *HMOX1*, encoding heme oxygenase 1 (HO1). The primer sequences were listed in **Table 1**. We used SYBRGreen PCR MasterMix (Thermo Fisher Scientific) was used to perform qPCR in the ViiATM 7 Real-Time PCR System (Applied Biosystems). The qPCR data was extracted using ViiATM 7 software and the expression level of each gene was normalized to the expression of the housekeeping gene - *ACTB*.

### Western Blot (WB) Assay

The bone tissues of interest were dissected and immersed in liquid nitrogen to snap freeze, which was followed by mechanical grinding using a mortar and pestle. Radioimmunoprecipitation (RIPA) lysis buffer was added and then incubated on ice for 20 minutes. The lysates were then centrifuged at 14,000 rpm for 25 minutes at 4 °C. The pellets were discarded, and the supernatants were collected as protein. Protein concentrations were determined using Bicinchoninic Acid (BCA) Protein Assay Kit (Beyotime) following the manufacturer's instructions. The protein extractions were separated by SDS-polyacrylamide gel electrophoresis (SDS-PAGE), which was then transferred to nitrocellulose membrane. The membrane was blocked using 5% skim milk (prepared in 1 x TBST) for 1 hour at room temperature (RT) under slow rocking. The primary antibodies diluted in 1% skim milk (in 1 x TBST) were added to the membrane for overnight incubation at 4 ℃. On the following day, membranes were rinsed with 1XTBST (5 minutes each time for 3 times). Primary antibodies for β-actin (sc-47778), RANKL (sc-377079), cathepsin K (sc-48353), γ-GCSc (sc-390811), and SOD1 (sc-101523) were purchased from Santa Cruz Biotechnology (Dallas, CA, USA). Primary antibodies for catalase (#14097S) and HO-1 (#70081S) were purchased from Cell Signaling Technology (Danvers, MA, USA). Appropriate anti-mouse or rabbit secondary antibodies conjugated with horseradish peroxidase (HRP) were used to incubate the membrane at RT for 1 hour. The proteins were detected using enhanced chemiluminescence substrate (PerkinElmer, Waltham, MA, USA). Images of protein bands were captured using an Image-quant LAS 4000 (GE Healthcare). The intensities of protein bands were analyzed using an ImageJ software (NIH, USA).

### Micro-CT scanning

Rat femurs were collected, and the excess soft tissues dissected. The femoral heads were scanned in a Skyscan Micro-CT instrument (Bruker) using parameter as the following: source current, 385 μA; source voltage, 65 kV; pixel size 9 μm; filter, AI 1.0 mm; rotation step, 0.4 degree. The image reconstruction was performed using NRecon software (Bruker) and the data was analysed using the CTAn program (Bruker). A refined volume of interest was generated 0.5 mm above the growth plate of the femoral head and 0.25 mm in height. The subchondral region of interest (ROI) within this volume was manually defined and determined using a constant threshold (50 ~ 255) for binarization of the trabecular bone. The parameters of the subchondral bone, including bone volume per tissue volume (BV/TV), trabecular number (Tb.N), trabecular separation (Tb.Sp), and degree of anisotropy (DA) were compared between the control group and ONFH model group.

### Statistical Analysis

All numeric data are presented as mean ± standard deviation (SD). Statistical difference was determined using Student's t test. The result was considered significantly different when p-value is less than 0.05.

## Results

### Imaging of late-stage of human ONFH

**Figure 1:**
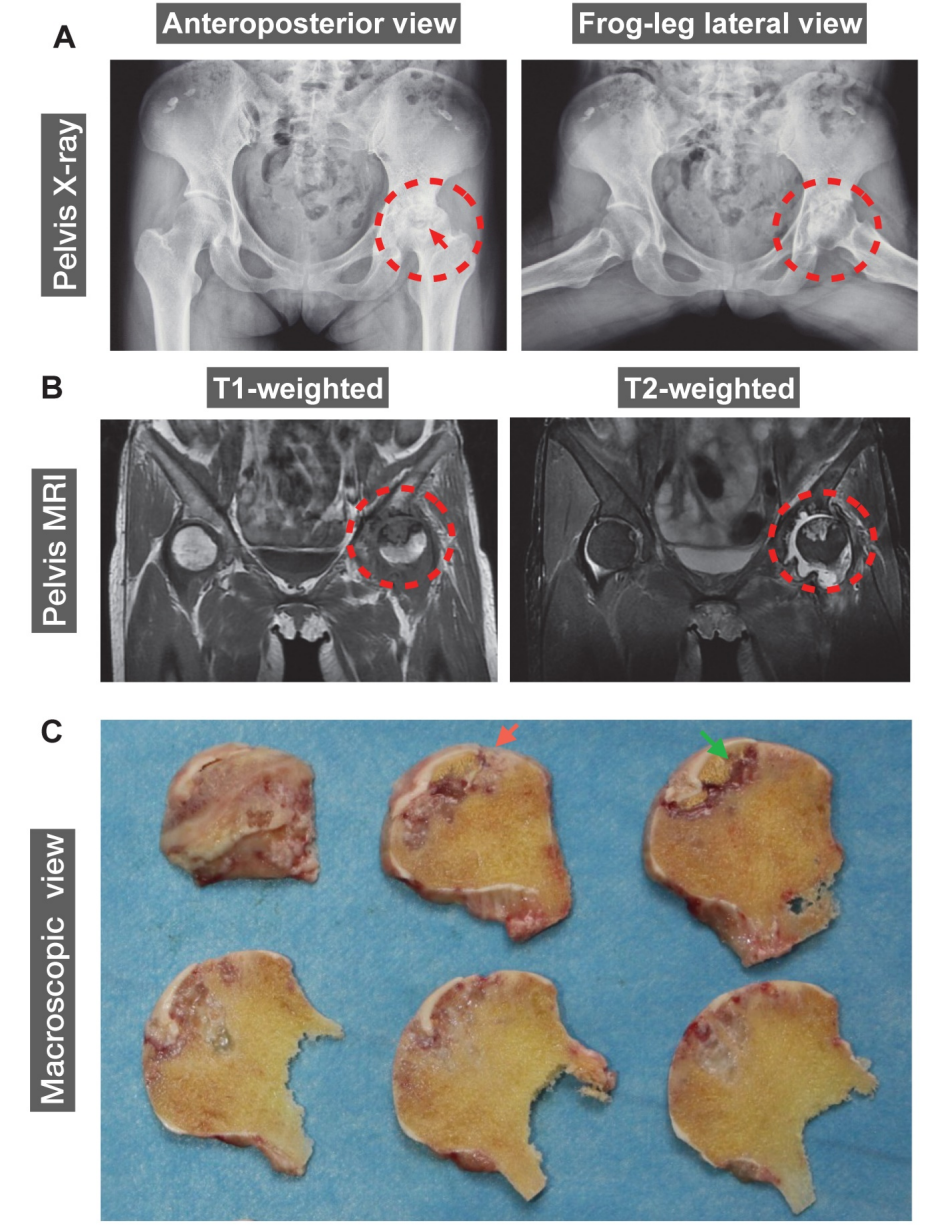
** A representative 42-year-old female case of steroid-induced osteonecrosis of femoral head (ONFH, ARCO stage IV). (A)** Radiographs delineating the crescent sign (red arrow), flattening of the articular surface of femoral head, and osteoarthritic acetabular changes (red dashed circles). **(B)** Coronal T1- and T2-weighted magnetic resonance images (MRI) highlighting a medium-sized necrotic lesion of the left femoral head (red dashed circles). **(C)** Gross appearance of sequentially sliced sections of the femoral head obtained at surgery, showing the deformation of the femoral head (red arrow) and subchondral necrotic bone (green arrow). ARCO, Association Research Circulation Osseous.

The human ONFH was diagnosed using X-ray and magnetic resonance imaging (MRI). A 42-year-old female patient with SLE for 20 years was also diagnosed with renal failure for 9 years. Steroids were given orally or intravenously since SLE diagnosis in 2000. Peritoneal dialysis or haemodialysis was performed periodically. The patient reported a complaint of left hip joint pain and was then diagnosed with ONFH in 2011. ONFH was gradually progressed to a late stage which required a THA. Representative anteroposterior and frog-leg lateral radiographs of the pelvis of this patient demonstrated a crescentic lucency in the subchondral area of left femoral head (crescent sign) (**Figure 1A**), suggesting the separation of subchondral bone from the overlying cartilage due to collapse of the femoral head. Joint space narrowing with acetabular involvement was also clearly delineated in **Figure 1A**, based on which the patient was diagnosed as stage IV according to Association Research Circulation Osseous (ARCO) staging system 24. Furthermore, MRI indicated the presence of a large-sized osteonecrotic lesion of the left femoral head, which presented as a low signal intensity area on T1-weighted image and a high signal on T2-weighted image (**Figure 1B**). The sequentially sliced macroscopic sections of the femoral head specimens clearly showed altered femoral head shape and osteonecrotic bone within the subchondral area (**Figure 1C**).

### Assessment of osteoclast activity and antioxidant enzymes in the necrotic bone of human femoral head

**Figure 2:**
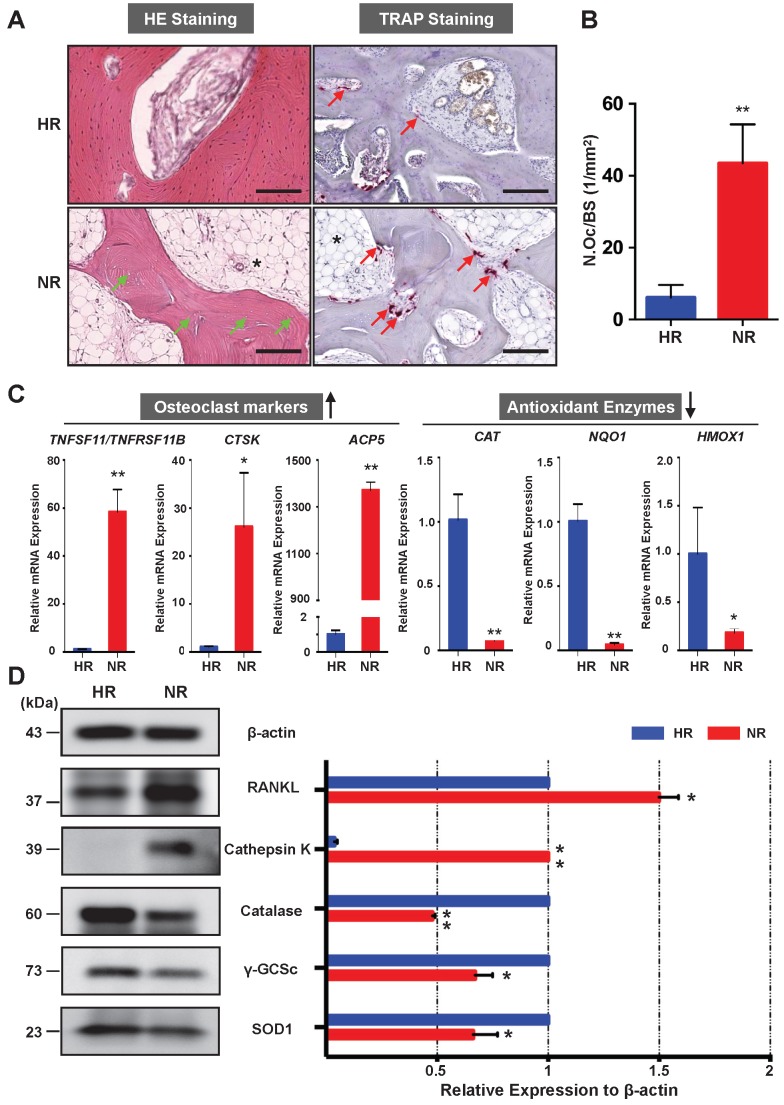
** Necrotic area of the femoral head has enhanced osteoclast activity and decreased expression of antioxidant enzymes. (A)** Hematoxylin & Eosin (HE) staining showing the necrotic bone is featured by the presence of empty osteocyte lacunae (green arrows) and tartrate-resistant acid phosphatase (TRAP) staining showing the distributions of osteoclasts on trabecular bone surface (red arrows) in the HR and NR. The accumulation of fat tissue (asterisk) is also notable in the NR. **(B)** Quantitative analyses of N.OC/BS. **(C)** qPCR results showing the genes' expression of osteoclast-specific markers and antioxidant enzymes relative to the *ACTB* expression. *TNFSF11*, encoding receptor activator of nuclear factor kappa-Β ligand (RANKL); *TNFRSF11B*, encoding osteoprotegerin (OPG); *CTSK*, encoding cathepsin K; *ACP5*, encoding TRAP; *CAT*, encoding catalase; *NQO1*, encoding NAD(P)H quinone dehydrogenase 1; *HMOX1*, encoding heme oxygenase 1 (HO1). **(D)** Western blot analysis showing the protein expression level of osteoclast-related markers including RANKL and cathepsin K, as well as anti-oxidant enzymes including catalase, γ-glutamylcysteine synthetase (γ-GCSc), and superoxide dismutase 1 (SOD1). All bar graphs are presented as mean ± SD (n=3 in each group). *P<0.05, **P<0.01 compared with control group (HR). HR, Healthy region; NR, Necrotic region; N.Oc/BS, osteoclast number/bone surface

The osteonecrotic bone tissues of the same patient were further analysed by histopathological evaluation. H&E staining of decalcified femoral head sections showed the uniform absence of osteocytes within lacunae, indicative of osteonecrosis in the necrotic region (**Figure 2A**). TRAP staining revealed that the necrotic bone tissues were widely circumscribed by osteoclasts (**Figure 2A**). The number of osteoclasts in the necrotic region was significantly higher than that in the healthy region (**Figure 2B**). It is also notable that there was an increase of fat tissue accumulation in the necrotic region as compared with the healthy region (**Figure 2A**). To assess for osteoclast activity and explore its relationship with oxidative stress, qPCR was performed to compare the difference of associated gene expression between healthy region and necrotic region (**Figure 2C**). Osteoclast-specific genes, including *CTSK* and *ACP5*, were robustly up-regulated in the necrotic region compared with those in the healthy region. The ratio of *TNFSF11* (encoding RANKL)/*TNFRSF11B* (encoding OPG) was also increased in the necrotic regions. In contrast, genes encoding anti-oxidant enzymes including *CAT* (encoding catalase), *NQO1* (encoding NAD[P]H quinone dehydrogenase 1), and *HMOX1* (encoding heme oxygenase 1 [HO-1]), were notably down-regulated in the necrotic bone. Furthermore, the western blot analyses of the osteoclast-related proteins (RANKL and cathepsin K) and antioxidant enzymes including catalase, γ-glutamylcysteine synthetase (γ-GCSc), and superoxide dismutase 1 (SOD1) further corroborated the mRNA findings, showing similar trends at the protein level (**Figure 2D**). The consistent expression trends of antioxidant enzymes and osteoclast markers were also noted in the other two cases. Together, these findings demonstrate that enhanced osteoclast activity is a pathological feature associated with ONFH which may, in part, due to the dysregulation of antioxidant enzymes and subsequent high oxidative stress.

### Micro-CT based analyses of trabecular microstructure of rat femoral heads

**Figure 3:**
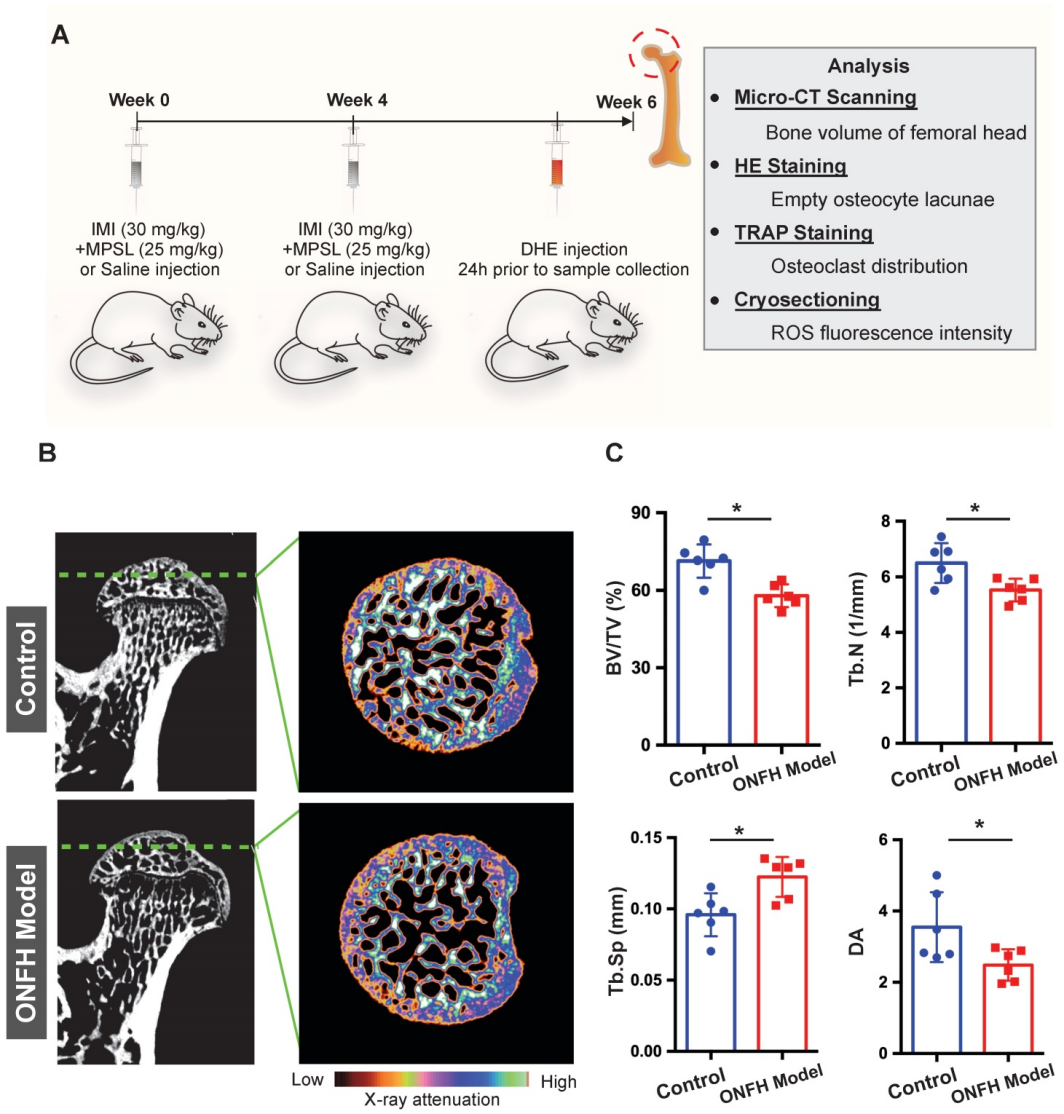
** Steroid-induced osteonecrosis of femoral head (ONFH) of rat and micro-CT (μCT) analysis. (A)** Schematic illustration of the establishment of rat ONFH model.** (B)** Representative μCT scanning images of the femoral head of the control group and model group. **(C)** Quantification of the bone microstructure parameters of the femoral head, including bone volume/tissue volume (BV/TV), trabecular number (Tb.N), trabecular separation (Tb.Sp), degree of anisotropy (DA). All bar graphs are presented as mean ± SD (n=6 in each group); *P<0.05 compared with the control group; ns, no significance.

To further explore the relationship between osteoclasts and oxidative stress in steroid-induced ONFH, we used a rat model to mimic ONFH (**Figure 3A**). The incidence rate of ONFH was 75% (6/8) in the model group with no ONFH observed in the control group. Micro-CT was used to examine the subchondral trabecular architecture and bone structural integrity of the femoral head. As was shown in **Figure 3B**, the bone microstructure in the ONFH model group was notably altered following the use of steroid. Furthermore, the quantification of the parameters including BV/TV, Tb.N, and Tb.Sp in the ONFH model group were significantly inferior to those of the control group (**Figure 3C**), indicating the bone loss in the femoral heads of the ONFH model rats. The alteration of DA also demonstrated the interrupted bone structure following the administration of steroid (**Figure 3C**).

### Histomorphometric analysis of steroid induced ONFH rat model

**Figure 4:**
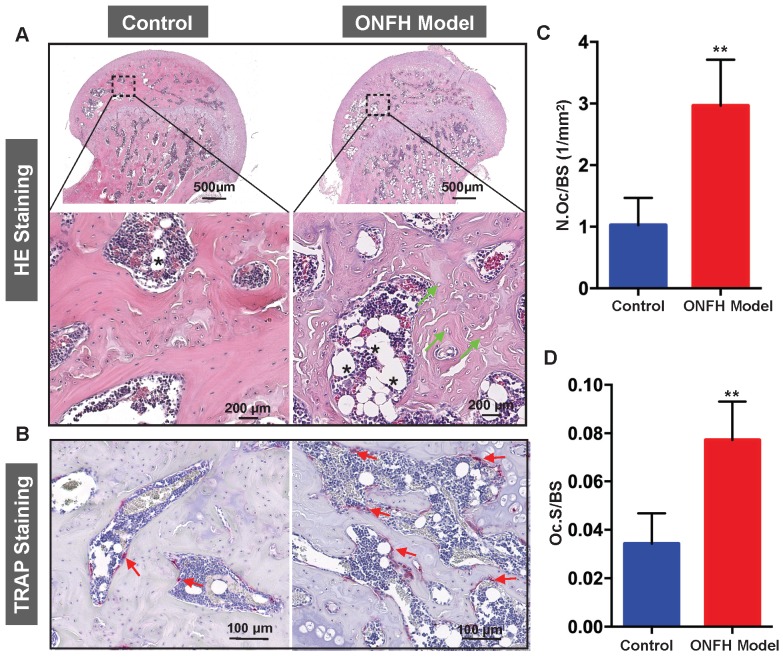
** Histomorphometric analysis of steroid-induced osteonecrosis of femoral head (ONFH) in rat model. (A)** Hematoxylin & Eosin (HE) staining of decalcified paraffin-embedding sections showing the increased number of empty lacunae (green arrows) and adipose tissue area (asterisk) in the model group. **(B)** Tartrate-resistant acid phosphatase (TRAP) staining showing the osteoclasts (red arrows) in the control and model groups. **(C and D)** Quantitative analyses of N.OC/BS and Oc.S/BS (N=5 per group). **P<0.01 compared with control group. N.Oc/BS, osteoclast number/bone surface; Oc.S/BS, osteoclast surface/bone surface.

To complement the studies, histomorphometric analyses were performed on decalcified sections of the femoral heads. From the H&E staining (**Figure 4A**), increased bone marrow adiposity and the presence of empty osteocyte lacunae were observed in the subchondral bone tissues, reminiscent of histopathological features observed in patient samples with ONFH. In addition, TRAP staining revealed a morphological increase in both osteoclast number (**Figure 4B and C**) and osteoclast surface (**Figure 4B and D**) surrounding the bone samples following steroid administration when compared with the osteoclasts in the control group.

### Detection of ROS and osteoclast-related markers in the rat femoral heads

**Figure 5:**
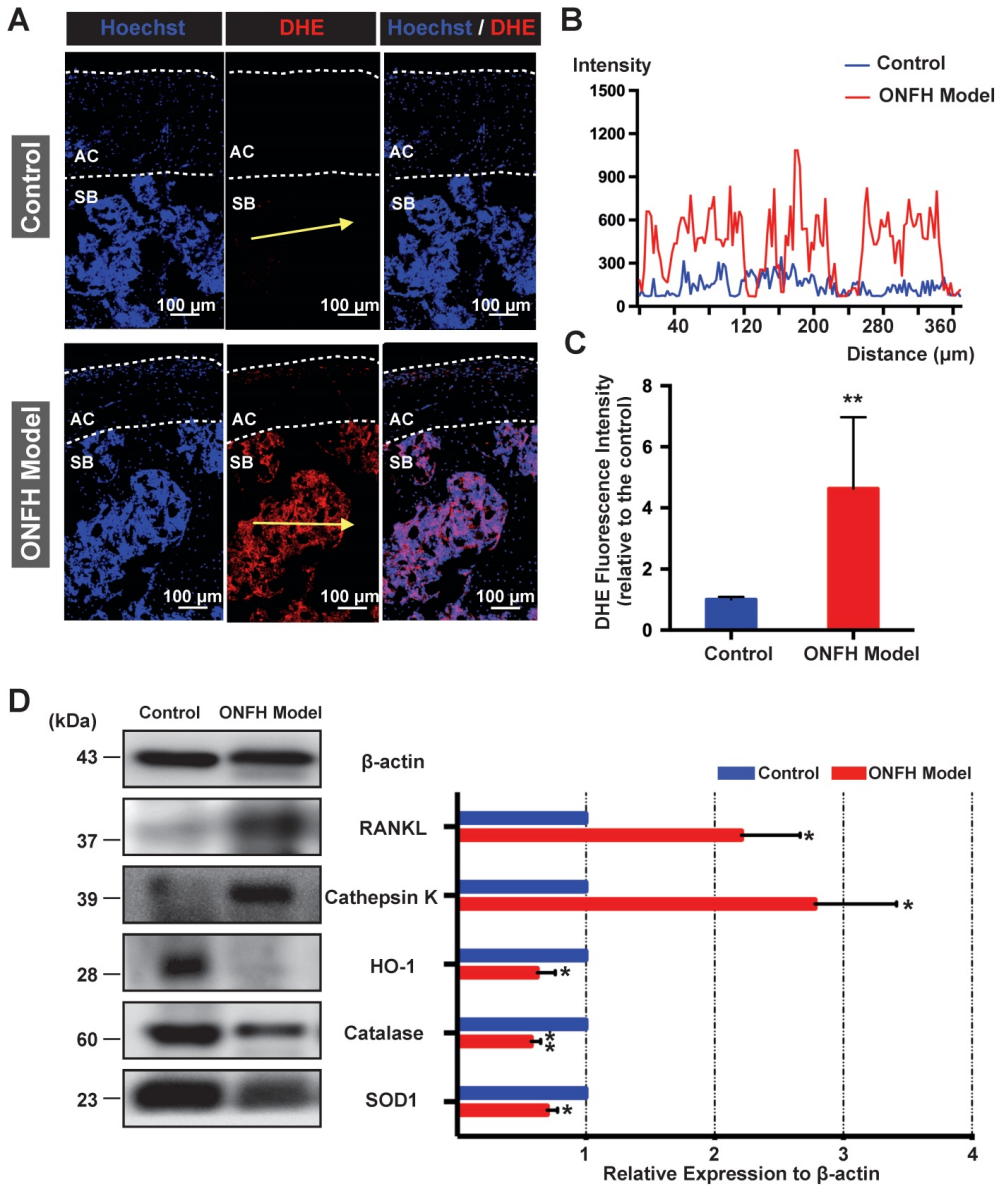
** Analysis of reactive oxygen species (ROS) level and antioxidant enzymes in the rat femoral head. (A)** Cryosections of femoral heads showing ROS level, as probed by dihydroethidium (DHE), in the control group and model group. AC, articular cartilage (white dashed line area); SB, subchondral bone. **(B)** DHE fluorescence intensity profile through subchondral area of femoral head (indicated by the yellow arrow in **A**) The abscissa of the DHE intensity profile represents the distance from the “start point” of the yellow arrow. **(C)** Quantification of DHE fluorescence intensity relative to the control group (n=5 in each group). **(D)** Western blot analysis of the protein expression level of osteoclast-related markers including RANKL and cathepsin K, as well as anti-oxidant enzymes including heme oxygenase 1 (HO1), catalase, and superoxide dismutase 1 (SOD1) (n=3 in each group). All bar graphs are presented as mean ± SD. *P<0.05, **P<0.01 compared with control group.

The *ex vivo* ROS level, as probed by DHE, was detected in the cryosections of femoral head under the confocal microscopy. As was shown in **Figure 5A**, DHE fluorescence of the subchondral area of the femoral head was dramatically enhanced in the model group. The fluorescence intensity profile of the yellow indicated line in the **Figure 5A** showed a clear difference between the control group and model group (**Figure 5B**). Quantification of the DHE fluorescence intensity indicated a higher oxidative stress in the rat femoral head following the steroid administration (**Figure 5C**). Western blot assay of the femoral head tissues further indicated that the expression levels of antioxidant enzymes such as HO-1, catalase, and SOD1 were significantly down-regulated in the model group (**Figure 5D**). In contrast, the expression of the mature osteoclast marker cathepsin K was upregulated in the ONFH model group (**Figure 5D**). In addition, the expression of RANKL was also increased (**Figure 5D**). Collectively, these results suggest that the use of steroid may lead to high oxidative stress in the femoral head by reducing antioxidant enzymes, which may contribute to the recruitment and/or activation of osteoclasts.

## Discussion

The use of steroids, particularly the prolonged and high-dose administration, is estimated to account for the majority of non-traumatic ONFH cases 4. ONFH is a progressive bone disorder which typically results in the collapse of the femoral head at end stage. The precise cellular and molecular mechanisms underlying steroid-induced osteonecrosis remain controversial. Recent studies indicate that increased osteoclast number and activity are responsible for the femoral head bone loss and subsequent failure 8, 10, 11. Given the increasing number of studies illustrating a role promising or ROS in promoting osteoclast formation and resorption, we sought to elucidate whether alterations of ROS level and osteoclasts lead to the pathogenesis of ONFH following the use of steroids. In the present study, ROS-related enzymes and osteoclasts were detected in both patients' ONFH specimens and rodent models mimicking ONFH. Specimens of femoral head were obtained from late-stage patients with progressive collapse of femoral head and required THA. Antioxidant enzymes and osteoclasts were then compared between healthy and necrotic regions. Steroid-induced ONFH rat models were then established to further assess the role of ROS and osteoclasts that played in this bone disorder.

Oxidative stress is featured by an augmented level of ROS which disrupts physiological reduction-oxidation (redox) balance. ROS are known to play a pivotal role in aging and degenerative diseases 25, 26. The deleterious effects of ROS result from the alternations to the integrity of mitochondrial and nuclear DNA, which can lead to cell apoptosis or necrosis 27. Accumulating studies have suggested that bone pathophysiology is closely related with redox balance and ROS are key modulators of bone cell functions 28. High oxidative stress is thought to be associated with the development of various bone disorders, including osteoporosis 29, 30, bone tumours 31, diabetic osteopenia 32, rheumatoid arthritis 33, and ankylosing spondylitis 34. Recent evidence also indicated that ROS are involved in the pathogenesis of steroid-induced ONFH 20, 21, 35 and that approaches inhibiting oxidative stress exert potential therapeutic effects on ONFH 35-37. To validate this, a ROS probe (DHE) was administrated on the rats prior to sacrifice, which is distributed via the circulation enabling the direct visualization of ROS in tissues as we previously described 18. Not surprisingly, the ROS fluorescence intensity in the microenvironment of subchondral area in femoral head was dramatically enhanced following steroid treatment. This is, to the best of our knowledge, the first time that the ROS levels have been directly visualized in the femoral head ex vivo. However, we are unable to identify the specific source of ROS, which may also be partly contributed by the altered conditions of osteocytes and fat tissues.

Oxidative stress is determined by the imbalance between ROS generation and its scavenging, the latter of which is carried out by well-known antioxidant enzymes such as SOD, HO-1, catalase, and γ-GCSc 38. The mechanism underlying the steroid-induced oxidative stress in the femoral head 39 remains unclear, but is thought that the administration of steroid leads to the downregulation of cytoprotective enzymes and subsequently causes redox failure 40, 41. Indeed, our results confirmed that the antioxidant enzymes (catalase, γ-GCSc, and SOD1) were significantly altered in the necrotic area of patients' femoral head relative to the healthy area, suggesting the existence of redox failure and the high level of ROS in the necrotic area. SOD plays a major role in cellular redox by reducing superoxide radicals to hydrogen peroxide (H_2_O_2_) which is less unstable 38. Catalase then converts H_2_O_2_ into oxygen and water, thereby protecting the cells from ROS damage 42. Furthermore, in addition to high ROS signalling in the femoral head, rats received steroids injection also showed decreased expressions of antioxidant enzymes including HO-1, catalase, and SOD1. A previous study demonstrated that the steroid administration may affect the antioxidant enzymes site-specifically, which was featured by an increased expression level of antioxidant enzymes such as SOD and catalase in liver, kidney, and heart as compared with humerus and femur 40. Following the use of steroid, the oxidation-reduction homeostasis in bone is compromised and therefore the bone was thought to be more susceptible to oxidative stress. These findings collectively suggest that the compromised expressions of antioxidant enzymes are, at least partly, responsible for the oxidative stress due to steroid administration.

The current understanding of the hazards of ROS is that oxidative stress may lead to pathological conditions, including the elevated vascular permeability 43 as well as DNA oxidation injuries 39 which cause cell apoptosis or necrosis 37. However, whether ROS enhance the osteoclast activity during the pathogenesis of ONFH has not been previously reported. ROS can support the activation of RANKL-induced signalling, which is essential for osteoclast formation and resorptive activity 15, 18, 44. The exact molecules or proteins that ROS target remain unclear, but RANKL-induced downstream events such as MAPK 15 and NF-κB signaling 45 appear to be involved. Our results demonstrate that the reduced antioxidant enzymes in the necrotic area are accompanied by the augmented osteoclast-related markers, such as cathepsin K, which were also supported by the TRAP staining indicating that osteoclasts activity is localized to the necrotic area. However, more clinical specimens as well as the investigation of mechanisms underlying the declined expression of the antioxidant enzymes are still needed in further study. Given the increased accumulation of fat tissue in the necrotic region, the loss of bone structure integrity could also be partly due to the impaired osteoblast differentiation. The corticosteroid treatment could increase adipogenesis by activating peroxisome proliferator-activated receptor gamma (PPARγ), but decrease osteogenesis, which was well documented previously 36, 46, 47. Consistent with these human specimens, we found that osteoclast numbers were elevated in rat models of steroid-induced ONFH, which ultimately resulted in decreased femoral trabecular bone volume as assessed by μCT (BV/TV, Tb.N, Tb.Sp). Relative to the control group, rats of the ONFH group also showed structurally disorganized bone as examined by the measurement of anisotropy. These results indicate that oxidative stress is closely related with the osteoclast activity and thus may coincide with the rapid progression of ONFH.

However, there are also some limitations in this study. Firstly, an anti-ROS group was not included in this study. It was previously indicated that anti-oxidative stress treatments indeed prevent or alleviate steroid-induced ONFH by affecting blood vessel, osteocytes, and adipose tissues 35, 36, 48-50, but with the lack of data on the role of osteoclasts. Osteoclast acts as a major contributor to the collapse of femoral head and the agents like bisphosphonates were also shown to have beneficial effects on ONFH 13. Herein, anti-ROS agents may also exhibit therapeutic effects by inhibiting excessive osteoclasts in steroid-induced ONFH, which needs to be further determined. Secondly, the outcomes or pathogenesis of steroid-induced ONFH in animals of different species 51 or/and in human may be variable. The human samples were collected from late-stage ONFH, while the animal models used in this study were more likely to be at early stage which may not coincide with the results on clinical perspective. Currently, rat models are still considered as desirable and suitable preclinical models to study molecular mechanisms of metabolic diseases 46. In this study, we proposed that ROS may activate osteoclasts and subsequently lead to the progression of ONFH. To address this hypothesis, early-stage rat ONFH seems well-represent the steroid-induced alterations of ROS and osteoclasts before the femoral head collapse. On the other hand, given the hard access to the human specimens at early stage, more advanced animal models which can mimic the late-stage ONFH are still required to be developed in the future.

In summary, this study demonstrates that a decline in the expression of antioxidant enzymes correlates with the administration of steroids, which may, in turn, contribute to elevated ROS levels and osteoclast numbers/activity observed during the pathogenesis of ONFH. Oxidative injuries usually present shortly following steroid administration at the very early-stage ONFH 39, 48, and excessive osteoclast activity is also believed to be a contributor for femoral head collapse. This provides rationale that antioxidant therapy may be a promising alternative to prevent the progression or collapse of steroid-induced ONFH by suppressing ROS level and thus inhibiting osteoclasts.

## Figures and Tables

**Figure 6:**
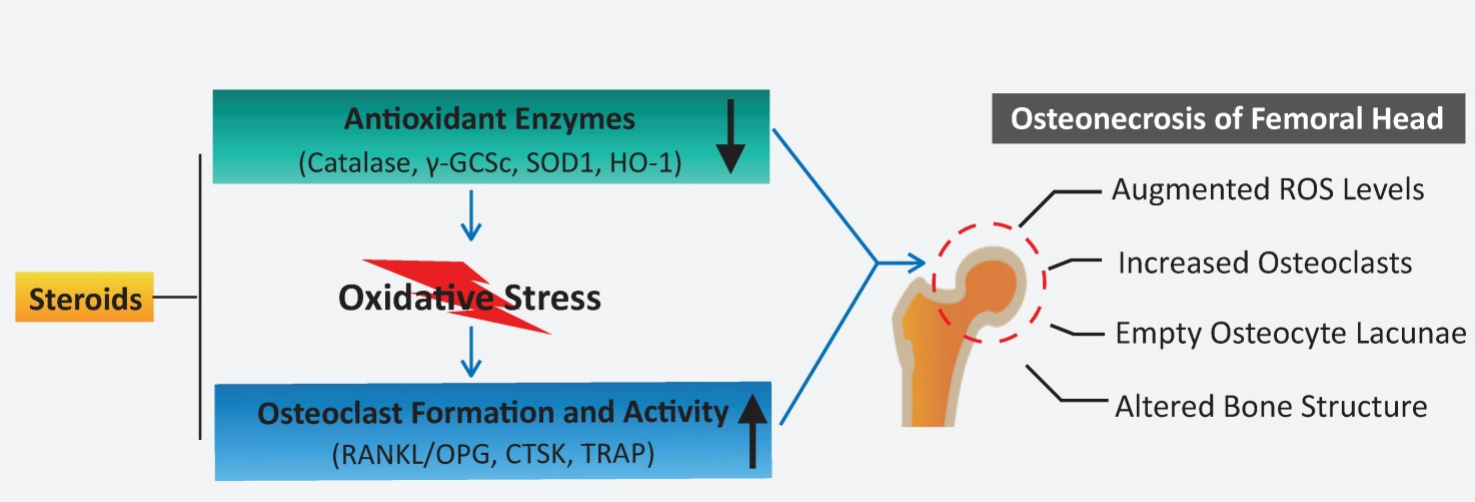
** A proposed scheme for enhanced oxidative stress and hyperactive osteoclasts following the use of steroids.** A decline in the expression of antioxidant enzymes correlates with the administration of steroids, which may, in turn, contribute to elevated ROS levels and subsequent osteoclast numbers/activity observed during the pathogenesis and progression of ONFH. HO1: heme oxygenase 1; ROS: reactive oxygen species; RANKL: receptor activator of NF-κB ligand; TRAP: tartrate-resistant acid phosphatase; γ-GCSc: γ-glutamylcysteine synthetase.

**Table 1:** Primers sequences

Genes	Forward Sequence (5'-3')	Reverse Sequence (5'-3')
*TNFSF11*	CATGTTCGTGGCCCTCCTG	GGATCCATCTGCGCTCTGAA
*TNFRSF11B*	GCGCTCGTGTTTCTGGACAT	ACACGGTCTTCCACTTTGCT
*CTSK*	GGGGGACATGACCAGTGAAG	CAGAGTCTGGGGCTCTACCT
*ACP5*	GGGAGATCTGTGAGCCAGTG	TTTATTCCCTCCCTGCCTGC
*CAT*	CTCCGGAACAACAGCCTTCT	ATAGAATGCCCGCACCTGAG
*NQO1*	GCTGGTTTGAGCGAGTGTTC	CTGCCTTCTTACTCCGGAAGG
*HMOX1*	CTGCTGACCCATGACACCAA	GGGCAGAATCTTGCACTTTGT
*ACTB*	ACAGAGCCTCGCCTTTGCC	GATATCATCATCCATGGTGAGCTGG

## Abbreviations

ARCO: Association Research Circulation Osseous
BCA: Bicinchoninic Acid
BV/TV: bone volume per tissue volume
CAT: catalase
CT: computed tomography
DA: degree of anisotropy
DHE: dihydroethidium
HO1: heme oxygenase 1
IMI: imiquimod
MMLV: moloney murine leukemia virus
MPSL: methylprednisolone
MRI: magnetic resonance imaging
NQO1: NAD[P]H quinone dehydrogenase 1
OCT: optimum cutting temperature
ONFH: osteonecrosis of the femoral head
OPG: osteoprotegerin
PFA: paraformaldehyde
PVP: polyvinylpyrrolidone
qPCR: Quantitative real-time polymerase chain reaction
ROS: reactive oxygen species
RANKL: receptor activator of NF-κB ligand
RIPA: radioimmunoprecipitation
SLE: systemic lupus erythematosus
SOD1: superoxide dismutase 1
THA: total hip arthroplasty
Tb.N: trabecular number
Tb.Sp: trabecular separation
TRAP: tartrate-resistant acid phosphatase
γ-GCSc: γ-glutamylcysteine synthetase
